# Stabilization of Urinary MicroRNAs by Association with Exosomes and Argonaute 2 Protein

**DOI:** 10.3390/ncrna1020151

**Published:** 2015-09-14

**Authors:** Cristina Beltrami, Aled Clayton, Lucy J. Newbury, Peter Corish, Robert H. Jenkins, Aled O. Phillips, Donald J. Fraser, Timothy Bowen

**Affiliations:** 1 Department of Nephrology, Wales Kidney Research Unit, School of Medicine, College of Biomedical and Life Sciences, Cardiff University, Heath Park, Cardiff CF14 4XN, UK; E-Mails: c.beltrami@bristol.ac.uk (C.B.); newburyl@cf.ac.uk (L.J.N.); jenkinsrh2@cf.ac.uk (R.H.J.); phillipsao@cf.ac.uk (A.O.P.); fraserdj@cf.ac.uk (D.J.F.); 2 Section of Oncology and Palliative Medicine, Institute of Cancer and Genetics, School of Medicine, College of Biomedical and Life Sciences, Velindre Hospital, Heath Park, Cardiff CF14 2TL, UK; E-Mail: claytona@cf.ac.uk; 3 BBI Group, The Courtyard, Ty Glas Avenue, Cardiff CF14 5DX, UK; E-Mail: PeterCorish@the-bbigroup.com

**Keywords:** microRNAs, urine, biomarker, chronic kidney disease, exosome, AGO2

## Abstract

A pressing need for new chronic kidney disease (CKD) biomarkers persists. MicroRNAs (miRNAs) are emerging as a novel class of disease biomarkers in body fluids, but mechanisms conferring their stability in urine have not been fully elucidated. Here we investigated stabilization in human urine of ubiquitously expressed miR-16, and miR-192, which we have shown previously to be downregulated in renal fibrosis, by association with extracellular vesicles and with argonaute protein (AGO) 2. Endogenous urinary miR-16 was significantly more resistant to RNase-mediated degradation than exogenous, spiked-in, *Caenorhabditis elegans* cel-miR-39. We used our previously optimized high-resolution exosome isolation protocol with sucrose gradient ultracentrifugation to sub-fractionate the primary extracellular vesicle-rich urinary pellet. MiR-16 and miR-192 were enriched in exosomal sucrose gradient fractions, but were also detected in all other fractions. This suggested association of urinary miRNAs with other urinary extracellular vesicles and/or pellet components, complicating previous estimates of miRNA:exosome stoichiometry. Proteinase K digestion destabilized urinary miR-16 and we showed, for the first time, RNA-immunoprecipitation of urinary miR-16:AGO2 and miR-192:AGO2 complexes. Association with exosomes and AGO2 stabilized urinary miR-16 and miR-192, suggesting quantitative urinary miRNA analysis has the potential to identify novel, non-invasive CKD biomarkers.

## 1. Introduction

Chronic kidney disease (CKD) is a major challenge to global health with serious implications for health and economic output [1,2]. The present diagnostic and prognostic test for intrinsic renal disease, biopsy, has a 3% risk of major complications. A potential end-point for numerous causal mechanisms, CKD is frequently accompanied by proteinuria. However, the predictive value of current non-invasive prognostic indicators such as urine protein quantification is limited since changes only become apparent following disease onset. There is, thus, a pressing need for new CKD biomarkers.

Powerful RNA detection techniques are now available for analysis of body fluids, but endogenous RNase activity degrades large transcripts, such as mRNAs. MicroRNAs (miRNAs) are short, endogenous, single-stranded noncoding RNA transcripts that inhibit target gene expression by translational repression and/or mRNA degradation. Quantitative analysis of miRNAs from body fluids, including urine, offers an alternative approach for the prognosis and/or diagnosis of various disorders [3,4,5] including CKD [6,7], diabetic nephropathy [8], renal fibrosis [9], tissue injury [10], cardiovascular disease [11,12], and cancer [13,14].

Much recent interest has focused on the association of body fluid miRNAs with extracellular vesicles, particularly exosomes, as a means of miRNA stabilization, as vectors for intracellular signaling by miRNAs, and as potential disease biomarkers [15]. Controversy remains, however, regarding the reproducibility of extraction and detection of exosomally-associated miRNAs from urine [16] and other body fluids [17], and whether limited miRNA:exosome stoichiometry obviates signaling [17].

Mature miRNA synthesis involves association with RNA induced silencing complex (RISC) proteins including argonaute proteins 1–4 (AGO1–4). Of these, miRNA:AGO2 association stabilizes plasma miRNAs [15,18] but AGO2 association with urinary miRNAs has not been investigated.

MiR-16 is highly expressed in cells comprising the various nephron regions [19] and was therefore selected for urinary analysis. We have previously demonstrated association of miR-192, one of the most highly expressed miRNAs in the kidney, with fibrosis in diabetic nephropathy [20] and investigated miR-192 disease-related mechanisms in the renal proximal tubular epithelial cell [21,22].

In the present study, we demonstrated the stability of endogenous urinary miR-16 and identified two stabilization mechanisms for miR-16 and miR-192. Since the role and biomarker utility of exosome-associated miRNAs in body fluids remain controversial, we isolated urinary exosomes using our previously optimized ultracentrifugation protocols [23], and demonstrated exosomal association of miR-16 and miR-192. We also showed, for the first time, association of urinary miR-16 and miR-192 with AGO2 protein. Our findings therefore suggest that quantitative analysis of urinary miRNAs offers a promising approach to the identification of novel biomarkers.

## 2. Results

### 2.1. Endogenous Urinary MiR-16 Is Resistant to RNase Digestion

**Figure 1:**
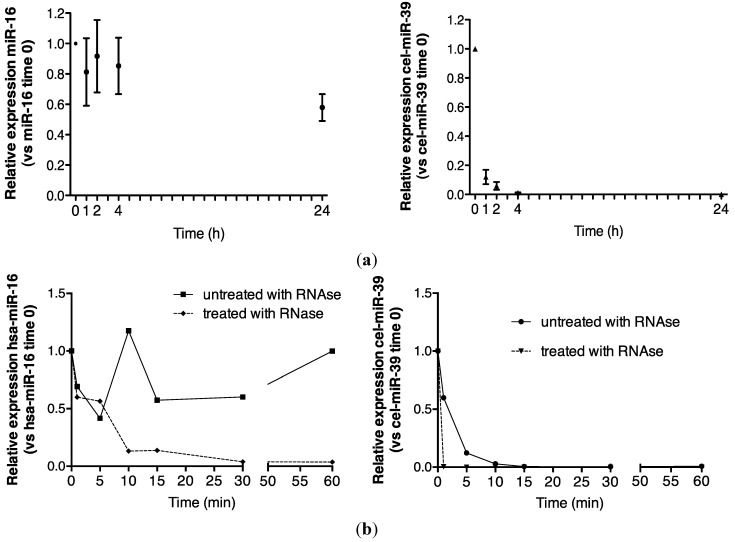

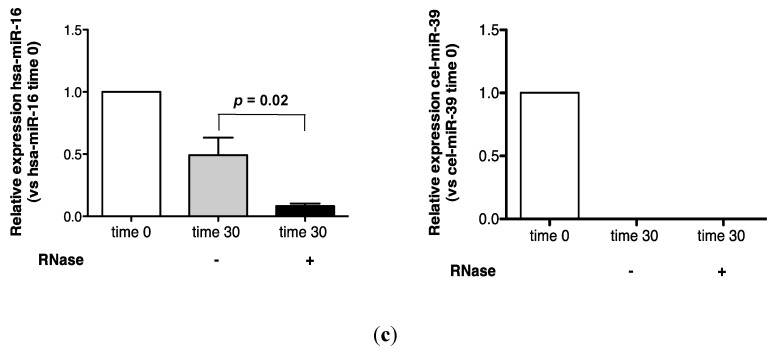
Urinary miR-16 is resistant to degradation by endogenous and exogenous RNAses. (**a**) Relative expression of endogenous miR-16 and exogenous cel-miR-39 in urine samples stored at room temperature over 24 h (*n* = 5), (**b**) following treatment with exogenous RNAse A over 1 h (*n* = 1) and (**c**) following 30 min of RNase A treatment (*n* = 5). Relative expression was calculated throughout using the 2^−∆∆CT^ method [24]. Where appropriate, data are expressed as mean values ± SEM.

Endogenous miRNA stability was evaluated by analyzing miR-16, a ubiquitously highly expressed transcript that was readily detected in urine in the present study. A comparison of detection of endogenous control miR-16 and spiked-in, exogenous, synthetic cel-miR-39 is shown in Figure 1a. While more than 50% of the time zero miR-16 signal was detected by quantitative reverse transcription polymerase chain reaction (RT-qPCR) analysis after 24 h, cel-miR-39 was rapidly degraded to <10% of its time zero signal after 1 h (Figure 1a).

Urinary miR-16 recovery was stable over 1 h in the absence of RNase A, while cel-miR-39 was wholly degraded after 15 min (Figure 1b). In the presence of RNase A, approximately 15% of the time zero miR-16 signal was detected at 15 min, while no cel-miR-39 was detected after 1 min (Figure 1b). Significantly increased miR-16 sensitivity to 30 min of RNase A digestion was observed (*p* = 0.02, Figure 1c).

Endogenous urinary miR-16 was markedly more stable than exogenous cel-miR-39, suggesting stabilization by specific mechanisms.

### 2.2. Association of Urinary MiR-16 and MiR-192 with Exosomes

**Figure 2:**
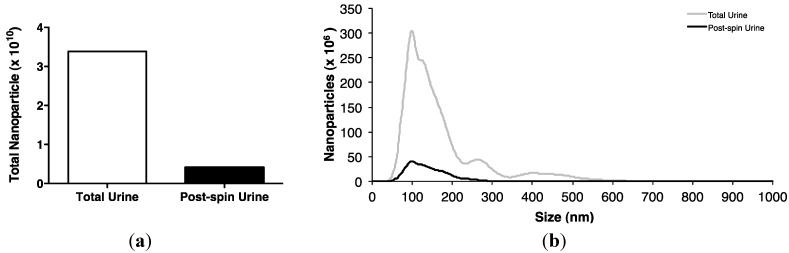
Isolation of urinary exosomes. (**a**) Ultracentrifugation of a pooled urine sample of 300 mL from 6 control subjects for 1 h at 200,000 g at 4 °C pelleted approximately 88% of urinary nanoparticles. (**b**) NanoSight LM10 analysis shows that most urinary nanoparticles were recovered between 70–200 nm with a peak at 100 nm, central to the size range typical of exosomes.

MiRNAs are present in exosomes: Spherical membrane-bound microvesicles of approximately 30–150 nm, purified from body fluids [15,16,17,23,25]. However, the contribution of this component to the total miRNA body fluid complement, and its functional significance, remain controversial [15,17,26,27]. Ultracentrifugation pelleted nearly 90% of urinary nanoparticles (Figure 2a), the majority of which were shown by nanoparticle tracking to be recovered between 70–200 nm, with the peak at 100 nm, central to the predicted size range typical of exosomes (Figure 2b).

**Figure 3:**
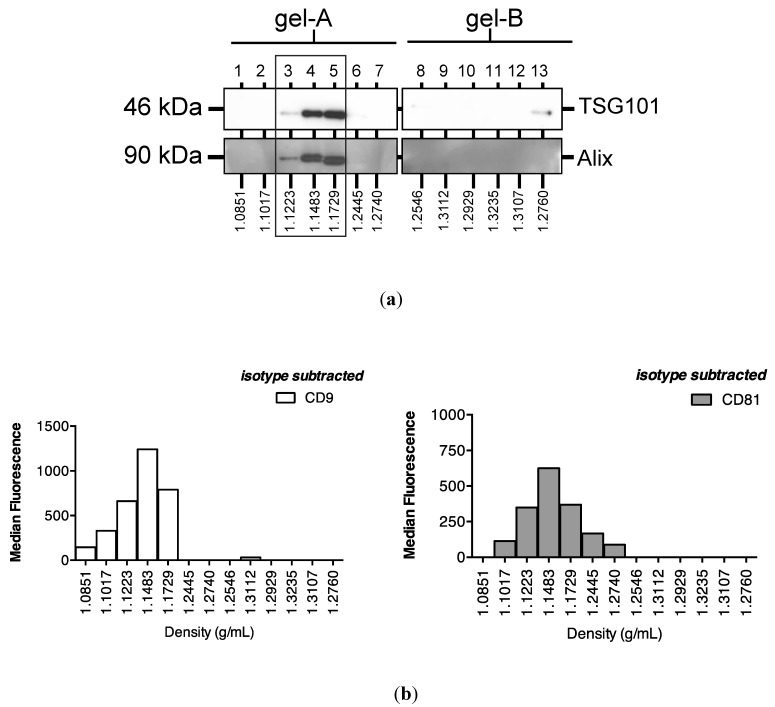

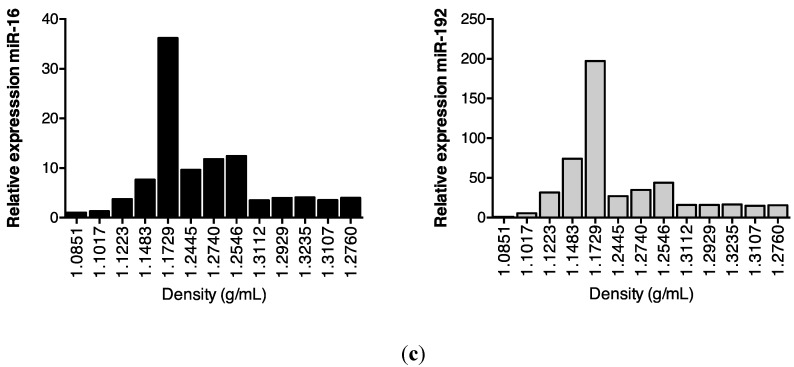
Enrichment of urinary miR-16, miR-192 and classical exosomal markers in the same sucrose gradient fractions. Following overnight sucrose gradient ultracentrifugation of the sedimented urinary pellet at 210,000 g at 4 °C, enrichment was observed in sucrose gradient fractions 3–5 of (**a**) multivesicular body markers TSG101 and Alix detected by Western analysis (**b**) exosome-expressed tetraspanins CD9 and CD81 detected by flow cytometry and (**c**) RT-qPCR-detected miR-16 and miR-192.

Subsequent sucrose gradient ultracentrifugation of the pellet isolated a series of sub-fractions, including those within the density range of 1.1–1.2 g/mL classically associated with exosomes [28]. These included fractions 3–5, in which Western analysis identified tumour susceptibility gene (TSG)101 and programmed cell death 6-interacting protein (Alix), markers of multivesicular bodies (Figure 3a). These fractions also contained an abundance of classical exosomally-expressed tetraspanins CD9 and CD81 (Figure 3b), as well as miR-16 and miR-192, both of which were consistent with exosomal association. However, Figure 3c also shows that miRNAs detected outside these exosome-enriched fractions, suggesting association of urinary miRNAs with other urinary extracellular microvesicles and/or pellet components.

Previous data have demonstrated the sensitivity of plasma-borne miRNAs to proteinase K [15]. However, since extracellular vesicle-associated miRNAs are proteinase K resistant [29], we therefore investigated the effect of proteinase K digestion on urinary miRNA stability.

### 2.3. Protein Association Stabilizes Urinary MiR-16

**Figure 4:**
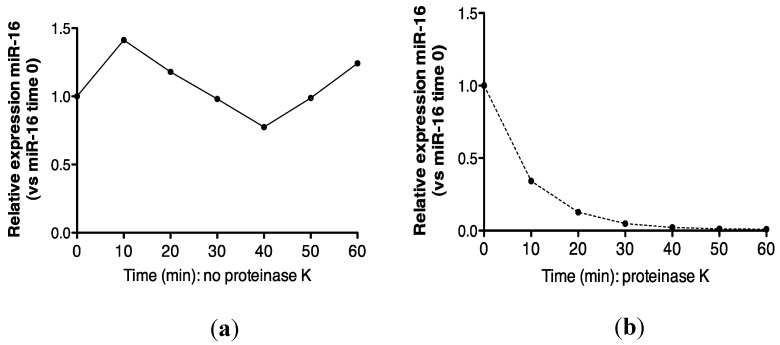

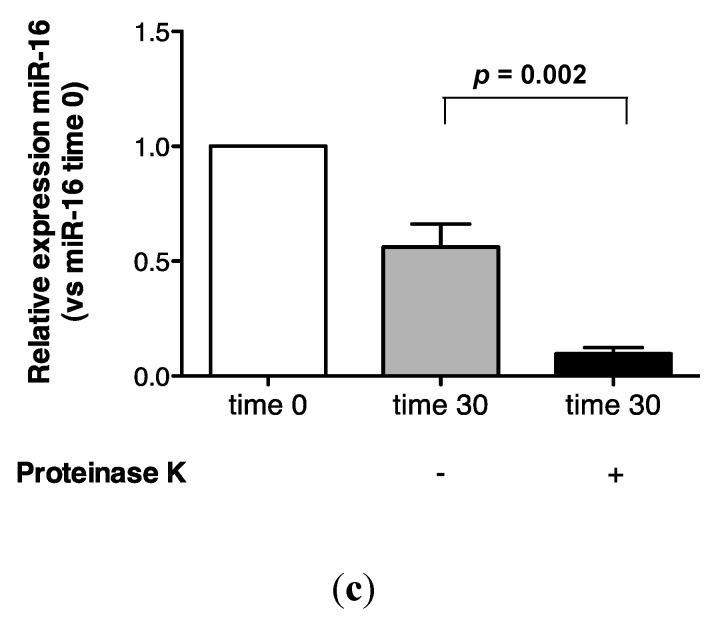
Proteinase K digestion at 55 °C sensitizes urinary miR-16 to degradation by endogenous RNases. (**a**) Relative expression of endogenous miR-16 in untreated urine (*n* = 1). (**b**) Relative expression of endogenous miR-16 in urine treated with proteinase K over 1 h (*n* = 1). (**c**) Relative expression of endogenous miR-16 following 30 min of proteinase K treatment (*n* = 5). Where appropriate, data are expressed as mean values ± SEM.

Recovery of miR-16 was compared in urine incubated at 55 °C alone, or in the presence of proteinase K. As shown (Figure 4a,b), approximately 100% recovery of miR-16 was obtained after 1 h at 55 °C, but this transcript was not detected following proteinase K digestion. Further analysis at the 30 min time-point showed that this effect was statistically significant (*p* = 0.002, Figure 4c).

MiRNA maturation involves association with the multi-protein RISC proteins AGO1 and AGO2 [15,18,30] in plasma and cultured cells, so next we analyzed miRNA:AGO2 association in urine by RNA-immunoprecipitation (RNA-IP).

### 2.4. Urinary MiR-16 and MiR-192 Associate with AGO2

**Figure 5:**
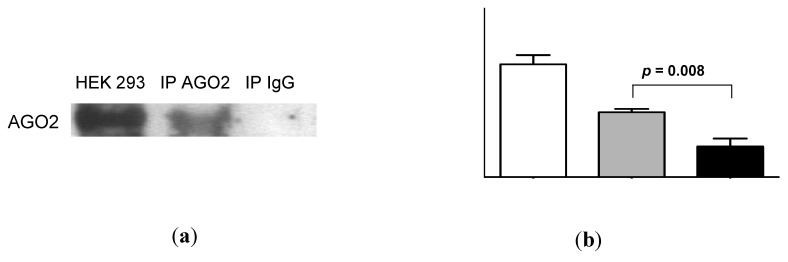

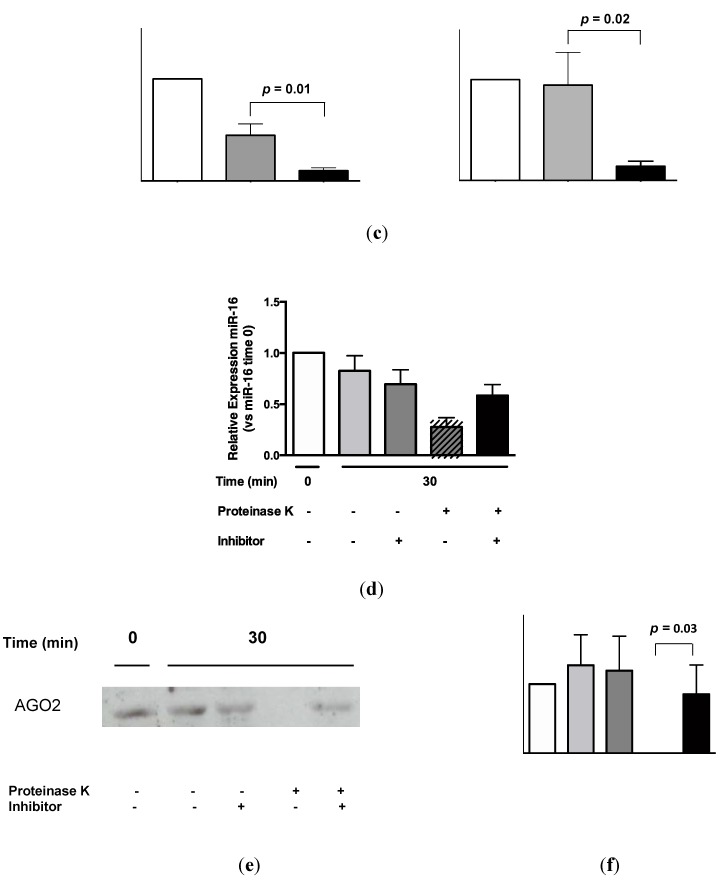
Urinary miR-16 and miR-192 associate with AGO2, while both miR-16 and AGO2 are protected from degradation following protease inhibitor treatment. (**a**) Western analysis of urinary AGO2 together with negative normal mouse IgG and positive HEK-293 controls. One representative gel is shown from a total of 5 experiments, each of which yielded similar results. (**b**) Analysis of densitometry data from Western blots (*n* = 5). (**c**) Detection of miR-16 and miR-192 by RNA-immunoprecipitation (*n* = 5). (**d**) Relative expression of miR-16 following 30 min of proteinase K treatment in the presence or absence of protease inhibitor PMSF (*n* = 8). (**e**) Western analysis of urinary AGO2 following 30 min of proteinase K treatment in the presence or absence of PMSF. One representative gel is shown from a total of 4 experiments, each of which yielded similar results. (**f**) Analysis of densitometry data from Western blots (*n* = 4). Where appropriate, data are expressed as mean values ± SEM.

AGO2 was readily detectable in urine by Western analysis (Figure 5a). Quantification of these data by densitometry showed a significant difference between urinary AGO2 and mouse immunoglobulin (Ig)G immunoprecipitation data (*p* = 0.008, Figure 5b). Subsequent RNA-IP analysis showed significant association of miR-16 and miR-192 with AGO2 (*p* = 0.01 and *p* = 0.02, respectively; Figure 5c). As shown in Figure 5d, the decrease in miR-16 detection following addition of proteinase K (*p* = 0.0002; see also Figure 4) was significantly attenuated in the presence of protease inhibitor phenylmethanesulfonylfluoride (PMSF, *p* = 0.003). Western analysis (Figure 5e) showed attenuated AGO2 detection in the presence of proteinase K, but AGO2 was detected following addition of both proteinase K and PMSF. Quantification of these Western data showed that this PMSF-mediated protection of AGO2 from proteinase K-mediated degradation was statistically significant (*p* = 0.003, Figure 5f).

## 3. Discussion

The principal findings of the present investigation were stabilization of urinary miR-16 and miR-192 by association with exosomes and AGO2.

Endogenous urinary miR-16 was resistant to RNase degradation in comparison with exogenous, spiked-in cel-miR-39. Like other body fluids, human urine contains active ribonucleases [31]. However, unlike mRNAs and long noncoding RNAs, plasma-borne miRNAs are resistant to endogenous RNase activity [13,15,26,32].

In HeLa cells, miR-16 is stable over 12 h, but miRNA stability may vary as a consequence of cell-/context-specific mechanisms [33,34]. Protection from degradation may be due to association with extracellular vesicles such as exosomes [11,35]. Exosomal transfer of functional miRNAs has been shown in mast cells [35] and dendritic cells [36]. In the healthy kidney, the constant passage of renal filtrate from glomerulus to collecting duct provides a potential route for exosomal miRNA transfer between different nephron regions [37].

However, previous analyses of human plasma have suggested that most miRNAs are not extracellular vesicle-associated [15,26]. More recent analyses of plasma, seminal fluid, dendritic cells, mast cells, and ovarian cancer cells have supported this assertion, positing that miRNA:exosome stoichiometry is too low to mediate signaling [15,17,26]. By contrast, it has also been proposed that miRNAs in serum and saliva are primarily concentrated in exosomes [27], and suggested that previous studies [15,26] underestimated exosomal miRNA abundance due to lowgrade vesicle lysis during extraction [27].

Mindful of this controversy, to demonstrate exosomal association of miR-16 and miR-192, we isolated urinary exosomes using protocols that we have verified previously. This included a step to further purify the sedimented ultracentrifugation pellet by a second ultracentrifugation step not used in several other studies [23]. By these means, we also detected significant abundances of miR-16 and miR-192 in other urinary extracellular vesicles and/or pellet components. Our observations concur with those from a recent comparison of methods for extracellular vesicle isolation from tissue culture medium [38]. At face value, the greatly reduced detection of miR-16 following RNase treatment might reflect that the majority of these mature transcripts detected were not protected within extracellular vesicles [29]. Further work will therefore be required for a detailed analysis of urinary exosomal miRNA content and transport in intra-nephronic signaling.

Plasma miRNAs that are not associated with extracellular vesicles may be stabilized by complex formation with RISC proteins AGO1 [18] and AGO2 [15,18,26], and the data in this study support association of AGO2 with miR-16 and miR-192. AGO proteins are key components of the RISC and effectors of RNAi that incorporate mature miRNAs following processing of their respective primary transcripts. AGO2 also functions in an RNase-independent manner, post-transcriptionally enhancing expression of mature miRNAs at either the processing or stability levels [39]. Just as miRNAs associated with exosomes might mediate signaling [23,39], stabilized AGO2-associated miRNAs are also potential mediators of signaling within the nephron, and this will also be worthy of further investigation.

In the present study, we showed that stabilization of endogenous urinary miR-16 was abrogated by proteinase K treatment. In addition, for the first time in urine, we used RNA-IP to demonstrate that AGO2 is a binding partner for miR-16 and miR-192.

## 4. Conclusions

In the present investigation we have demonstrated two methods of miR-16 and miR-192 stabilization, supporting the hypothesis that urinary miRNAs have significant potential as CKD biomarkers. Conclusions on signaling in the nephron mediated by exosomal, extracellular vesicular and non-extravesicular urinary miRNAs will require further analysis.

## 5. Materials and Methods

### 5.1. Study Population

Urine samples were obtained from the Wales Kidney Research Tissue Bank and approval for the study granted by its governance committee. Mean and range values for 10 (7 male) unaffected control subjects: Age = 43.8 (25–60); urine protein (g/L) = 0.08 (0.07–0.14); urine pH = 5.6 (5–7).

### 5.2. Urine Collection and RNA Isolation

Second pass morning urine (20 mL) was collected, placed on ice and processed immediately. Samples were centrifuged at 2000 g for 10 min at 4 °C, and supernatants transferred to fresh, sterile universal tubes for RNA isolation.

RNA was isolated from all samples using the miRNeasy kit (Qiagen, Crawley, West Sussex, UK) with the following modifications to the manufacturer’s protocol. Carrier RNA (MS2 RNA; Roche Diagnostics Limited, Burgess Hill, West Sussex, UK) was added at 1 μg/750 μL of QIAzol reagent, prior to mixing with urine samples. The mixture was incubated at room temperature for 5 min, then 0.5 pM *Caenorhabditis elegans* miR-39 (MC10956, cel-miR-39; Life Technologies, Paisley, Renfrewshire, UK) was spiked into each sample, and the entire aqueous phase from each sample was loaded onto a single affinity column.

### 5.3. Reverse Transcription-Quantitative PCR (RT-qPCR) Analysis

Reverse transcription (RT) was carried out using the High-Capacity cDNA RT Kit (4368814, Life Technologies, Carlsbad, MA, USA). The RT master mix for one reaction was composed of: 4.25 μL of water, 1.5 μL of 10 × RT Buffer, 0.15 μL of 100 mM dNTP, 0.1 μL of 40 U/μL RNase Inhibitor (M0307S, New England BioLabs® Inc., Ipswich, MA, USA), 1 μL of 50 U/μL MultiScribe Reverse Transcriptase and 3 μL of 5 × RT-primer specific for each miRNA. Then, 10 μL of the RT master mix was added to 4 μL of water plus 1 μL of RNA for the urine samples. The RT non-template control (RT-NTC) negative control reaction contained an equal volume of water instead of RNA. The following thermal cycler profile was used: 30 min at 16 °C, 30 min at 42 °C, 5 min at 85 °C, followed by cooling to 4 °C. The cDNA was diluted with water 1:3, and 4 μL was used in qPCR. For each transcript, the master mix for each reaction was prepared by combining 1 μL of miRNA-specific set of PCR-primers and TaqMan probe (Life Technologies), 5 μL of water and 2 × Universal PCR Master Mix (4440047, Life Technologies). MiRNA-specific master mix (16 μL) was distributed to appropriate wells on an Optical 96-Well Fast Plate (Life Technologies) followed by 4 μL of pre-diluted cDNA, or water for NTCs. Plates were sealed with MicroAmp Optical Adhesive Film (Life Technologies) and qPCR was performed on a ViiA7 Real-Time PCR System (Life Technologies) using the manufacturer’s recommended cycling parameters: 10 min at 95 °C followed by 40 cycles of 15 s at 95 °C and 1 min at 60 °C. The following TaqMan assays were used: MiR-16, 000391; miR-192, 000491; cel-miR-39, 000200 (Life Technologies). Relative expression was calculated by standard means [24].

### 5.4. Room Temperature Urine Analysis over 24 h

Urine samples from 6 control subjects were incubated at room temperature over 24 h, and miRNAs were isolated from 250 μL aliquots after 0, 1, 2, 4 and 24 h, with 0.5 pM cel-miR-39 spiked into each sample at time zero.

### 5.5. RNase Treatment of Urine

Two 2.5 mL aliquots were taken from each of 5 urine samples from control subjects. In the negative control urine aliquot, 0.5 pM of cel-miR-39 and 250 μL of RNase storage buffer were added, the latter replaced by 0.1 mg/mL of RNase A (AM2272, RNase A (RPA Grade); Life Technologies (Ambion)) in the treated sample. Following incubation of 1 control sample in duplicate at 37 °C, aliquots of 250 μL were removed after 1, 5, 10, 15, 30 and 60 min to which 750 μL of QIAzol plus 1 μg of carrier RNA was added, and samples were then stored at −80 °C. RNA was isolated and miR-16 detected as described above. Following analysis, the experiment was then repeated at the 30 min time-point using 10 samples.

### 5.6. Proteinase K Treatment of Urine

Urine samples from 2 control subjects were incubated at 55 °C ±50 μg/mL of proteinase K (P-2308; Sigma-Aldrich, Gillingham, Dorset, UK). Aliquots of 250 μL were removed after 0, 10, 20, 30, 40, 50 and 60 min to which 750 μL of QIAzol plus 1 μg of carrier RNA was added, and samples were then stored at −80 °C. RNA was isolated and miR-16 detected as described above. Following analysis, the experiment was then repeated at the 30 min time-point with urine samples from 5 control subjects.

We then analysed proteinase K digestion in the presence of 5 mM phenylmethanesulfonylfluoride (PMSF), a serine protease inhibitor, in control urine samples. One half of each sample was concentrated using an Ambion-Ultra-0.5 mL centrifugal filter (NMWL 10 kDa; Millipore, UFC501008) prior to Western analysis for AGO2. The concentrated samples were mixed 1:3 with reducing buffer (50 mM Tris-HCl, 2% SDS, 20 mM DTT and 0.002% (*w*/*v*) bromophenol blue) and heated for 5 min at 95 °C before processing, as described below in Section 5.8. The other half was eluted in 750 μL of QIAzol plus carrier RNA (MS2 RNA, Roche) and processed for RNA isolation and miR-16 detection as described above.

### 5.7. AGO2 Immunoprecipitation

A 200 μL aliquot of Magna Bind goat anti-mouse IgG Magnetic Bead (Thermo Fisher Scientific, Waltham, MA, USA) was washed 3 times with PBS solution (300 μL), and incubated with 10 μg of mouse monoclonal anti-AGO2 (ab57113; Abcam, Cambridge, UK) or mouse IgG (Santa Cruz Biotechnology, Dallas, TX, USA) antibodies for 2 h at 4 °C. The preincubated beads and antibody were then added to 400 μL of urine and incubated overnight at 4 °C. Beads were washed 3 times with 1% Nonidet P-40 buffer (1% Nonidet P-40, 50 mM Tris-HCL, pH 7.4, 150 mM NaCl, 2 mM EDTA) and resuspended in 200 μL of PBS. One half of each of 10 control samples was eluted in loading buffer, following which 5 samples were analysed by SDS/PAGE and immunoblotting with rabbit polyclonal anti-AGO2 antibody (ab32381; Abcam) with a positive control extracted from human embryonic kidney (HEK)293 cells. The other half was eluted in 750 μL of QIAzol plus carrier RNA (MS2 RNA, Roche) and processed for RNA isolation and miRNA detection.

### 5.8. Western Blotting

Proteins were solubilised by the addition of reducing buffer (see Section 5.6). Samples were electrophoresed through 4%–12% Bis-Tris gels (Life Technologies) for exosome markers or 7.5% Polyacrylamide gels for AGO2 and transferred to Protran nitrocellulose membranes (GE Healthcare, Chalfont St Giles, Buckinghamshire, UK. Blots for exosome markers were blocked overnight (3% non-fat milk in PBS + 0.05% Tween 20) then probed with antibodies against classical exosome markers TSG101 and Alix (Santa Cruz Biotechnology via Insight Biotechnology, Wembley, Middlesex, UK). After 3 washes (PBS + 0.05% Tween 20) blots were stained with goat anti-mouse IgG-HRP conjugated antibody (1:15,000 in PBS + 0.05% Tween 20, Santa Cruz Biotechnology). Blots for AGO2 protein were blocked for 2 h (3% non-fat milk in PBS + 0.1% Tween 20) then probed overnight with rabbit polyclonal anti-AGO2 antibody at 4 °C (ab32381; Abcam 1:2000). After 3 washes (PBS + 0.05% Tween 20) blots were stained with goat anti-rabbit IgG-HRP conjugated antibody for 2 h at room temperature (RT) (1:10,000 in PBS + 0.1% Tween 20, Santa Cruz Biotechnology). Bands were visualized using the ECL+ system and photographic film (GE Healthcare).

### 5.9. Isolation of Sedimented MiRNAs

A pooled urine sample from control subjects with a total volume of 300 mL was centrifuged at 400 g for 10 min to pellet cells, followed by a further 2000 g for 10 min to remove cellular debris. The resultant supernatant was then centrifuged at 200,000 g for 1 h, after which the total sedimented pellet was resuspended in 100–150 μL of PBS and total protein quantified by micro-BCA protein assay (Thermo Fisher Scientific, CramLington, Northumberland, UK).

### 5.10. Separation of Extracellular Vesicles by Density Gradient Centrifugation

A total of 300 μg of total sedimented pellet (see above) was overlaid onto a continuous sucrose gradient of 0.2–2.5 M sucrose. Samples were centrifuged at 4 °C overnight at 210,000 g using an MLS-50 rotor in an Optima-Max ultra-centrifuge (Beckman Coulter, High Wycombe, Buckinghamshire, UK). The refractive index of collected fractions was measured at 20 °C in an automatic refractometer (J57WR-SV; Rudolph Research Analytical via Spectronic Camspec Ltd, Leeds, UK) and from this the density was calculated as described previously [40]. Fractions were washed in PBS by ultracentrifugation at 150,000 g in a TLA-110 rotor (Beckman Coulter) and pellets resuspended in a small volume before aliquoting into 3 different microtubes: MES buffer (0.025 M MES, 0.154 M NaCl, pH 5) was added to the first prior to coupling to microbeads for flow cytometric analysis [40], SDS loading buffer was added to the second prior to Western analysis, and the third was used for sedimented miRNA extraction.

### 5.11. Flow Cytometric Analyses of Exosome-Coated Beads

The assigned aliquot from above was coupled to 0.5 μL of stock beads (surfactant-free, aldehyde sulfate 3.9 μm beads, Life Technologies) that had been washed twice in MES buffer. Exosome beads were incubated in a final volume of 100 μL of MES buffer at room temperature for 1 h on a shaking platform, followed by rolling overnight at 4 °C. Beads were blocked by incubating with MES buffer containing 1% BSA for 2 h at room temperature. Blocking buffer was washed away, and beads were resuspended in MES buffer containing 0.1% BSA. Primary monoclonal antibodies (2–10 μg/mL) against CD9 (R&D Systems, Abingdon, Oxfordshire, UK) and CD81 (AbD Serotec, Kidlington, Oxfordshire, UK) were used for 1 h at room temperature. After one wash, goat anti-mouse Alexa Fluor 488-conjugated antibody (Life Technologies) diluted 1:200 in MES buffer containing 0.1% BSA was added for 1 h. After washing, beads were analyzed by flow cytometry using a FACS-Canto instrument configured with a high throughput sampling module running FACSDiva Version 6.1.2 software (BD Biosciences, Oxford, UK) and median fluorescence values were plotted.

### 5.12. Nanoparticle Tracking Analysis

Extracellular vesicles present in urinary samples were analyzed by nanoparticle tracking using the NanoSight LM10 system (NanoSight Ltd, Amesbury, Wiltshire, UK) configured with a 405 nm laser and a high sensitivity digital camera system (OrcaFlash 2.8; Hamamatsu C11440, NanoSight Ltd). Videos of 60 s were collected and analyzed using NTA-software (version 2.2) with the minimal expected particle size set to 30 nm, and minimum track length and blur set to automatic. Each sample was diluted in nanoparticle-free water (Fresenius Kabi, Runcorn, Cheshire, UK) to a concentration of between 2 × 10^8^ and 9 × 10^8^ particles/mL. This was carried out in triplicate for pre-ultracentrifuged urine, and for 4 replicates following 200,000 g ultracentrifugation.

### 5.13. Statistical Data

Data were analyzed using GraphPad Prism version 6 software to run the student’s *t*-test, or, for our PMSF data, a repeated measures one-way ANOVA with Bonferroni adjustment. Values for *p* below 0.05 were considered significant.
